# The value of a combined radiological–surgical approach in allowing curative resection of a locally advanced type IIIa Klatskin tumor

**DOI:** 10.1093/jscr/rjab033

**Published:** 2021-03-29

**Authors:** Marco Fronda, Damiano Patrono, Andrea Doriguzzi Breatta, Giulia Osella, Carlo Gazzera, Gianluca Paraluppi, Paolo Fonio, Dorico Righi, Renato Romagnoli

**Affiliations:** 1 Radiology 1U—Diagnostic Imaging and Interventional Radiology Department, AOU Città della Salute e della Scienza di Torino, Turin, Italy; 2 General Surgery 2U—Liver Transplant Center, AOU Città della Salute e della Scienza di Torino, Turin, Italy

## Abstract

We report the case of a 53-year-old patient subjected to percutaneous embolization of right and middle hepatic veins to induce liver segments 2–3 hypertrophy before extended right hepatic resection for a locally advanced type IIIa perihilar cholangiocarcinoma.

Hepatic vein embolization (HVE) was performed 3 weeks after surgical recanalization of left portal vein (severely narrowed at its origin due to tumor infiltration) interposing an internal jugular vein graft between main and distal left portal vein.

Nine days after HVE, future liver remnant volume increased from 395 to 501 cc, i.e. 25.1% of standardized total liver volume, allowing to perform a radical right hepatic trisectionectomy plus caudatectomy. He was discharged home on postoperative day 15th after an uneventful postoperative course, with no sign of posthepatectomy liver failure.

## INTRODUCTION

Surgical resection is the only potentially curative option in patients with locally advanced perihilar cholangiocarcinoma (Klatskin tumor). Curative resection of perihilar cholangiocarcinoma usually requires sacrificing a large portion of hepatic parenchyma, which, in association with cholestasis, increases the risk of posthepatectomy liver failure. Thus, a multidisciplinary strategy allowing radical resection leaving a functional future liver remnant (FLR) is crucial. In patients with normal hepatic function, FLR ranging from 20 to 30% of nontumor liver volume is generally considered to be safe [1].

Here, we present the case of a patient suffering from a complex type IIIa Klatskin tumor in which a staged radiological–surgical approach allowed resection with curative intent of a T4 tumor infiltrating proximal left portal vein [2].

## CASE REPORT

A 53-year-old male patient, 178 cm, 100 kg, presented with jaundice and bile ducts dilatation detected at ultrasound (US) examination.

**Figure 1:**
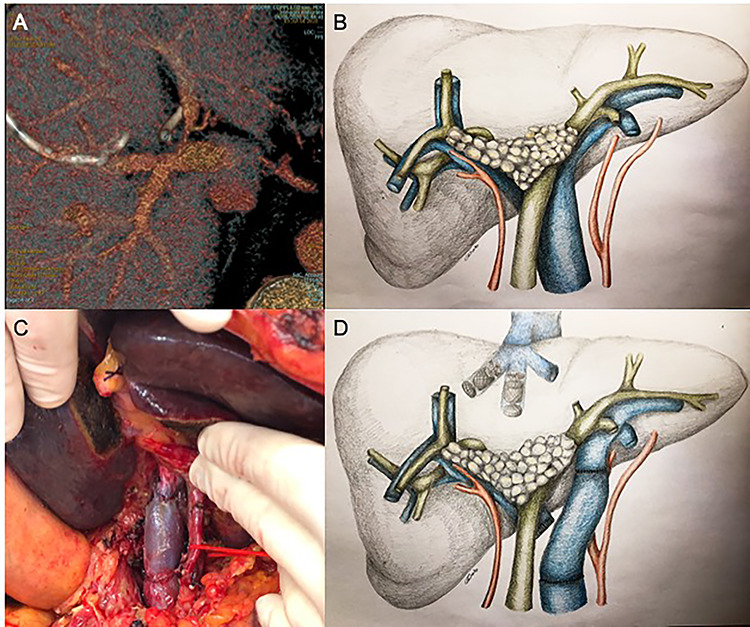
Since significant left portal vein narrowing at its origin due to tumor infiltration (**A**, **B**) contraindicated upfront right portal vein embolization, which was deemed potentially ineffective and at risk of favoring portal trunk thrombosis, porto-portal shunt by the mean of an autologous internal jugular vein graft interposition, followed by right and middle hepatic veins embolization, was performed (**C**, **D**).

Contrast-enhanced computed tomography (CECT) and magnetic resonance cholangiography revealed a type IIIa Klatskin tumor, closely adherent to portal vein bifurcation and infiltrating the proximal left portal vein. The stenosis was hemodynamically significant at US-Doppler examination (Fig. 1A and B).

In order to resolve jaundice, the patient underwent left percutaneous transhepatic biliary drainage with concurrent endoluminal biopsy, which confirmed the diagnosis of perihilar cholangiocarcinoma, and external biliary drainage on the right side.

The tumor was technically resectable by a right trisectionectomy with segment 1 and portal vein resection at this stage. However, FLR volume, as evaluated by CECT, was 395 cc, representing 14.9% of total liver volume (TLV) and 19.8% of standardized TLV (sTLV) [3], thus precluding upfront resection.

Given the important narrowing of the proximal left portal vein, which would have jeopardized any attempt at inducing FLR hypertrophy, the patient first underwent surgical left portal vein recanalization. This was achieved by interrupting main and left portal vein outside the infiltrated area (which was deliberately not touched) and interposing an autologous internal jugular vein graft; portal bifurcation was by-passed, and portal flow was completely diverted to the left hemi-liver (Fig. 1C).

Unfortunately, CECT performed 3 weeks after the operation showed no increase in FLR volume, with partial reperfusion of multiple small intrahepatic right portal branches, characterized by reversed flow. Considering the technical difficulty of portal vein embolization (PVE) in this setting, percutaneous embolization of the right and middle hepatic veins (HVE) was considered as a salvage strategy (Fig. 1D).

**Figure 2:**
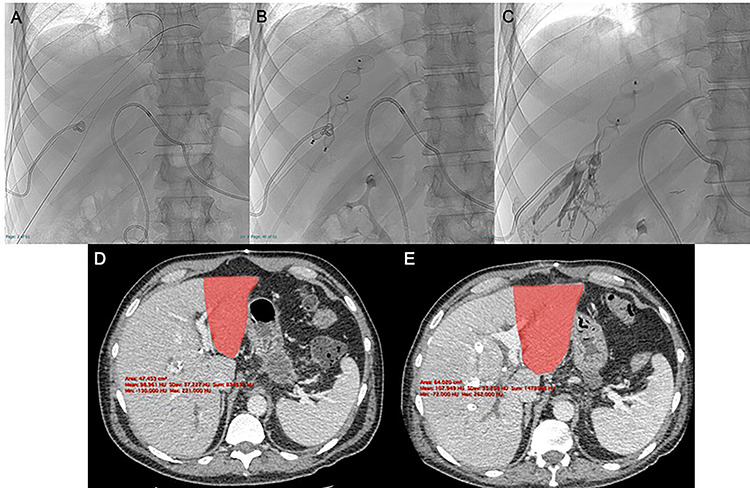
After US-guided percutaneous transhepatic access with micropuncture sets (**A**) the right and middle HVE was performed using two 90% oversized vascular plugs (**B**) and then completed with 2:1 Lipiodol-glue mixture (**C**), resulting in a 26.8% increase in volume of the FLR (501 vs. 395 cc) after 9 days with a kinetic growth rate (KGR) of 11.8 cc/days (**D**, **E**).

Under local anesthesia and conscious sedation, the right and middle hepatic veins (HVs) were accessed under ultrasonographic guidance with 21G Chiba needles, 0.018″ guidewires and micropuncture sets (Meditalia Biomedica, Medolla, Italy) (Fig. 2A).

Two 0.035″ guidewires (Amplatz Super Stiff, Boston Scientific, Marlborough, MA) were advanced to the atrio-caval junction and, after accommodation of two 8F × 24 cm sheaths (Radifocus Introducer II, Terumo, Japan), 6 and 7 mmHg of endoluminal pressure were measured, respectively, in the right and middle HVs. After that, both HV were occluded with Amplatzer Vascular Plugs II (St-Jude Medical, Plymouth, MN), respectively, 22 and 16 mm in diameter (90% oversized), with the distal part deployed 10–15 mm proximal to the junction with the inferior vena cava to enable further surgical ligation (Fig. 2B). The embolization of the HVs and potential collaterals was distally completed with a 2:1 mixture of Lipiodol Ultrafluid (Guerbet, Villepinte, France) and Glubran 2 (GEM Italy, Viareggio, Italy) (Fig. 2C).

The CECT scan performed 9 days after the HVE showed a 26.8% increase in volume of the FLR (501 vs. 395 cc), resulting in a kinetic growth rate (KGR) of 11.8 cc/day. FRL/TLV ratio was 20.4% (+5.5%) and the FRL/sTLV ratio was 25.1% (+ 5.3%) (Fig. 2D and E).

**Figure 3:**
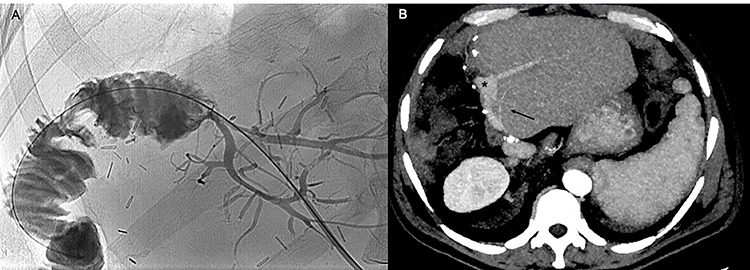
The cholangiogram performed 1 month after the hepatic resection (**A**) and the CT scan performed 5 months later (**B**) show regular patency of the bilio-enteric anastomosis, absence of biliary leaks and patency of the portal graft, the left portal branches (black asterisk) and left hepatic artery (black arrow), without signs of local recurrence.

Two weeks after HVE, patient underwent right hepatic trisectionectomy with caudatectomy and extra-hepatic bile ducts resection. During operation, temporary hepatic pedicle clamping during parenchymal transection was complicated by common hepatic artery thrombosis, which was managed by anastomosing the left hepatic artery to the sectioned stump of an aberrant right hepatic artery. Complete resection was confirmed by pathology report, revealing clear resection margins around a poorly differentiated bile duct adenocarcinoma with extensive venous/perihepatic fat and focal parenchymal infiltration, and micrometastasis in one hilar lymph node (pT4N1). Patient had an uneventful postoperative course and was discharged home on postoperative day 15th (Fig. 3A).

At 6-month follow-up, he is disease free and currently being administered adjuvant chemotherapy (Fig. 3B).

## DISCUSSION

Several strategies have been proposed to increase FLR volume, including PVE [4], associating liver partition and portal vein ligation for staged hepatectomy (ALPPS) [5] and sequential (HVE) following PVE [6]. More recently, liver venous deprivation (LVD), associating PVE and HVE in a single procedure [7], and extended LVD (eLVD—embolization of both right and middle HVs) [8] have proven to be safe and effective.

In the present case, FLR increase after HVE was comparable with that previously described for PVE alone^,^ [4] but faster, showing a KGR of 11.8 vs. 4.4 cc/days [9]. In terms of KGR, HVE was comparable with LVD (9.8 cc/days) [7], but inferior to ALPPS (25.4–32.7 cc/days) [9] and eLVD (25 ± 8 cc/days) [8]. It is worth noting that in our case the right portal vein had been previously sectioned.

The technique used for HVE was the same described by Guiu *et al*. [7, 8] for LVD and eLVD, except for some small arrangements. In particular, since previous portal flow diversion might have resulted in compensatory increased arterial flow to the right hemi-liver, we measured HVs pressure before plug placement. Furthermore, after verifying the absence of a significant overpressure, plugs were oversized by 90% to prevent their migration. For this purpose, an US-guided very peripheral percutaneous access to HVs was crucial.

The relevance of our case is in the association of an original surgical approach with HVE, which both concurred in determining sufficient FLR hypertrophy.

Whether liver volume can be considered a valid surrogate of liver function is debated.

HVE, alone or in association with portal vein ligation, needs to be further investigated in larger studies, evaluating its effectiveness on both FLR volume and function. Our experience suggests that in case of difficult PVE, HVE is a feasible and effective salvage strategy.
